# Development of a Combined 2D-MGD TLC/HPTLC Method for the Separation of Terpinen-4-ol and α-Terpineol from Tea Tree, *Melaleuca alternifolia*, Essential Oil †

**DOI:** 10.3390/biom15010147

**Published:** 2025-01-18

**Authors:** Aimé Vázquez, Nurhayat Tabanca

**Affiliations:** United States Department of Agriculture-Agricultural Research Service (USDA-ARS), Subtropical Horticulture Research Station (SHRS), Miami, FL 33158, USA; aime.vazquez@usda.gov

**Keywords:** tea tree oil, *Melaleuca alternifolia*, terpinen-4-ol, α-terpineol, HPTLC, 2D-MGD TLC, enantiomeric ratio, authentication

## Abstract

Tea tree oil (TTO), acquired from *Melaleuca alternifolia* (Maiden & Betche) Cheel, Myrtaceae, is a widely utilized essential oil (EO) due to its bioactive properties. The identification and quantification of TTO ingredients is generally performed by GC-MS, which provides the most accurate results. However, in some instances, the cost and time of analysis may pose a challenge. Thin-layer chromatography (TLC) and high-performance thin-layer chromatography (HPTLC) offer a simpler, faster, cost-effective alternative capable of simultaneously analyzing and quantifying multiple samples. In addition, for more complex oils, two-dimensional (2D) or multigradient development (MGD) TLC provide better separation. Nevertheless, further development is sometimes necessary for the isolation of comigrating components. This study showcases a combined 2D-MGD TLC/HPTLC method for the successful separation of TTO components of interest. While human error, limited separation, and the partial evaporation of volatile components may still present a challenge during the process, considerable recovery of mono- and sesquiterpenes was achieved. This protocol also resulted in the successful isolation of target oxygenated monoterpenes (OMs) producing highly pure terpinen-4-ol (100%) and α-terpineol (≥94%), confirmed by GC-MS. The accurate enantiomeric distribution of these major OMs was verified by GC-FID through the use of a chiral cyclodextrin-based stationary phase. The observed positive enantiomer range (area percent) as well as (+)/(−) ratio for each terpinen-4-ol and α-terpineol were within acceptable ISO criteria.

## 1. Introduction

Tea tree oil (TTO), an essential oil obtained from the native Australian tree *Melaleuca alternifolia* (Maiden & Betche) Cheel, Myrtaceae [1], is widely used in skin and hair care products, in addition to aromatherapy and medicine, due to its antimicrobial properties [2,3,4,5,6,7]. Furthermore, TTO and its components have been studied for their biological activity towards mosquitoes [8] and other agricultural pests, such as fruit flies [9,10,11,12,13,14,15], that cause great damage to a wide variety of crops worldwide. For this purpose, international organizations like the Food and Agriculture Organization (FAO) and the International Atomic Energy Agency (IAEA) have joined forces to create and implement mitigation protocols [16] for the management of invasive insect pests. Though highly efficient, limitations regarding the cost and availability of existing lures have prompted a search for novel, readily available, and cost-effective attractants, including essential oils such as TTO.

Quality characteristics of TTO components play a key role in therapeutic use and biological activity [17,18]. According to international standards, certified Australian TTO comprises 30–48% terpinen-4-ol, followed by γ-terpinene (10–28%) and α-terpinene (5–13%). Secondary components include α-terpineol, *p*-cymene, α-pinene, and terpinolene [17,19]. Due to the geographic location and extraction protocol, oil composition may slightly vary; yet, for commercial purposes, it shall remain within the established acceptable margins. Unfortunately, as a result of commercialization and demand, the adulteration of oils has also become a frequent problem. Until recent years, GC-FID and GC-MS have been the main techniques utilized for the authentication of TTO [18,20]. However, runtime and cost of operation of GC and GC-MS may become a disadvantage, prompting a search for a more viable alternative.

Traditional TLC [21,22] has served as a valuable screening tool for essential oil chemical composition under a variety of protocols. Using primarily a combination of toluene and ethyl acetate as the mobile phase, a guide was created that assists in the identification of possible TTO degradation products or adulterants on silica gel plates [23]. In addition to separation and identification, and due to its non-destructive nature, TLC also offers a means for the isolation and purification of individual components that may be utilized as reference standards or as samples for additional research. Notwithstanding, traditional TLC has its shortcomings, a critical issue being the intrinsic human error, which hinders reproducibility. Moreover, due to the limited surface area of interaction on a TLC plate, some samples may not separate properly, especially those containing a large number of components. Thus, further separation becomes necessary.

Manual unidimensional multigradient development (MGD) [24,25,26] provides additional separation in comparison to traditional TLC. However, though a highly useful technique, it also seems to work best on simple mixtures and lower sample concentrations. Manual two-dimensional (2D) TLC [27,28,29] also improves separation. Yet, despite having a second (perpendicular) development, components with similar retention factors (R_F_) may still prove to be difficult to isolate.

More effective methods for the separation of plant extracts, such as essential oils (EOs), and other complex mixtures, combine 2D with multigradient development (2D-MGD) TLC [30], as well as TLC coupled with other techniques [31]. In this case, the initial 1D TLC development is followed by a series of developments in order of polarity, while placing and keeping the plate at a 90° angle (perpendicular) from the original position. As much as TLC is still an efficient screening protocol for most EOs, human error and external conditions such as temperature and humidity are likely to cause diffusion or shifts in the retention factor and may generate considerable variation among results. As a direct consequence, automation has always been at the forefront of TLC research [32,33,34,35]. With the development of high-performance thin-layer chromatography (HPTLC), planar chromatography has evolved into a solid, dependable standardized technique accepted in pharmacopeias for the analysis of EOs and other natural products [36,37,38,39]. This software-based automated system is also compliant with GMP regulations [40,41,42] and several studies have coupled 2D-MGD with HPTLC automation for enhanced separation [43,44,45].

Other than percent composition, another important aspect of quality control of EOs is the ratio of enantiomers present since this is a unique trait of each oil. Several chiral chromatography methods have been used for the analysis of enantiomers [46,47,48,49]. In the case of TTO [50,51,52] and other EOs [53,54], protocols have been developed to establish authenticity. Davies et al. [55] also provided guidance based on measurements of 57 authentic tea tree oils (±3 standard deviations) by three independent laboratories using different GC-FID and GC-MSD methods.

Considering that TTO is a complex mixture, it is expected that some of its components might possess very similar R_F_ values. An example of this is terpinen-4-ol and α-terpineol, which are structurally similar and may prove to be difficult to isolate at high purity by traditional TLC. In this situation, a 2D-MGD TLC/HPTLC method would not only provide better separation and higher reproducibility but would also reduce the time required to identify active molecules in TLC/HPTLC-based bioassays, where compounds are difficult to separate by single-elution TLC.

This article proposes a novel protocol to isolate target TTO components in sufficient amounts to be utilized for further research. The described 2D-MGD TLC/HPTLC method in this article will prove to be useful and efficient on the separation of comigrating TTO compounds such as terpinen-4-ol and α-terpineol. Though with some limitations, the developed method will also aid in the separation of two large comigrating groups, monoterpene and sesquiterpene hydrocarbons. The enantiomeric distribution of terpinen-4-ol and α-terpineol was obtained by chiral GC-FID evaluation.

## 2. Materials and Methods

Recent manual TLC preparatory separations of TTO [56] were achieved following Wagner and Bladt’s traditional method for essential oils [23], except for the substitution of toluene/ethyl acetate at 93:7 (*v*/*v*) with hexane/acetone at 9:1 (*v*/*v*) as the solvent system. In that experiment, relatively large amounts of material were needed for further studies. Therefore, 150 μL of a 60% TTO solution was manually applied onto a thick silica gel plate with a capillary micropipette. Development and derivatization were also performed manually. One crucial limitation of manual TLC is the introduction of human error. In this case, components of interest appeared as large unresolved color zones difficult to properly isolate for follow-up studies.

Also, an observed disadvantage of acetone as a developing solvent is its possible interaction with sample components and with the stationary phase, which may generate oxidation byproducts. More so, the presence of acetone makes the solvent system more difficult to evaporate from the plate, resulting in the evaporation of the most volatile oil constituents and causing interference in the derivatization process.

As a baseline of TTO separation for our study, a chromatographic fingerprint of TTO at low concentration was then developed using HPTLC to provide individual color zones specific to TTO under more controlled conditions.

### 2.1. HPTLC Fingerprint/1D TLC

An automated HPTLC system (CAMAG, Muttenz, Switzerland) was used for the analysis, employing a variation in an existing method [41,56], to separate TTO constituents. However, for this study, toluene was replaced by n-hexane as part of the solvent system, later updated to cyclohexane in lieu of hexane [57].

#### 2.1.1. Sample and Plate Preparation

Two TTO samples (1%) were prepared by adding 0.1 mL of the corresponding essential oil (certified TTO from Apothecary Shoppe, Portland, OR, USA, and SAT Group, Kannauj, India, respectively) to 9.9 mL methylene chloride, CAS # 375-09-2 (Thermo Scientific, Fair Lawn, NJ, USA). Individual reference standards, diluted in methylene chloride in concentrations proportional to those in TTO [17], were also analyzed for R_F_ verification. Other *Melaleuca* oils were diluted to 1% in methylene chloride and included for comparison. A ready-to-use universal HPTLC mix (UHM) (Sigma-Aldrich Co., St. Louis, MO, USA) was used as the system suitability standard (SST) [58] (Table 1). The stationary phase was a 20 × 10 cm HPTLC Si 60 F_254_ plate (Merck, EMD Millipore Corp., Burlington, MA, USA) activated at 100 °C for 10 min.

#### 2.1.2. Development and Detection

A mobile phase comprising Supelco OmniSolv^®^ n-hexane, 95%, CAS # 110-54-3 (EMD Millipore Corp., Burlington, MA, USA), and HPLC-Grade ethyl acetate, 99.7%, CAS# 141-78-6 (Sigma-Aldrich Co., St. Louis, MO, USA), at a ratio of 8:2 (*v*/*v*) was utilized as the developing solvent.

A CAMAG ATS4 (CAMAG, Muttenz, Switzerland) autosampler, in the spray-on mode, was employed to apply samples as 8 mm bands, 11.4 mm apart, 8 mm from the bottom, and 20 mm from the left edge of the plate. Nitrogen gas was used to evenly spray the samples onto the plate at 250 nL/s. The application volume for each sample and reference standard was 1 μL. Twice the volume (2 μL) of UHM was applied. The plate was developed in a CAMAG ADC2 (CAMAG, Muttenz, Switzerland) chamber containing a saturation pad. Prior to development, the chamber was saturated with 25 mL of the mobile phase for 20 min. and activated for 10 min. with a saturated aqueous solution of magnesium chloride (MgCl_2_). Ten milliliters (10 mL) of the mobile phase was used for the chromatographic separation. Development was carried out up to 85 mm, followed by a 1 min air-drying period inside a fume hood.

**Figure 1:**
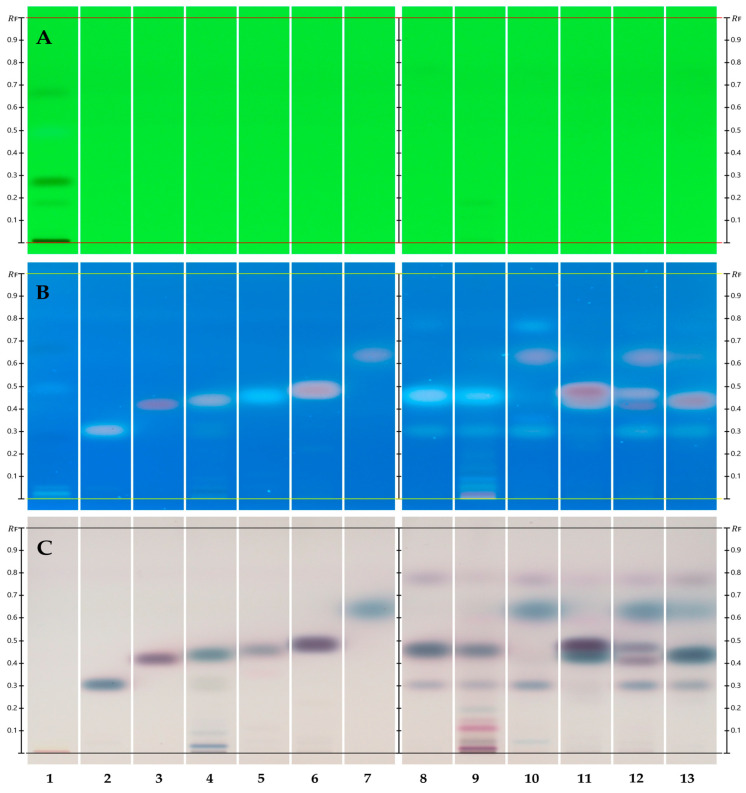
One-dimensional HPTLC separation of TTO components using n-hexane/ethyl acetate at 8:2 (*v*/*v*). (See Table 1 for track assignment.) Separation of SST before derivatization under shortwave UV light (254 nm) ((**A**) track 1). Reference standards and *Melaleuca* spp. EOs post derivatization under longwave UV (350 nm broadband) (**B**) and white light RT (**C**).

Derivatization occurred in a CAMAG derivatizer (CAMAG, Muttenz, Switzerland) equipped with a yellow nozzle set at spray level 3, and these settings continued to be used for all automatic derivatizations. A vanillin/sulfuric acid solution was used as the derivatizing reagent. A 10 mg/mL ethanolic vanillin solution was prepared by dissolving 1 g of vanillin, CAS # 121-33-5 (Sigma-Aldrich Co., St. Louis, MO, USA) in 100 mL of 96% ethanol, CAS # 64-17-5 (Decon Labs Inc., King of Prussia, PA, USA). To create the final reagent, 60 μL of concentrated sulfuric acid (H_2_SO_4_), CAS # 7664-93-9 (Fisher Scientific, Fair Lawn, NJ, USA), was added to 3 mL of ethanolic vanillin and the mix was evenly sprayed onto the developed plate. Colors were revealed by heating the derivatized plate on a CAMAG Plate Heater III (CAMAG, Muttenz, Switzerland) for 3 min. at 100 °C and images were taken in a CAMAG Visualizer 2 (CAMAG, Muttenz, Switzerland) (Figure 1).

**Figure 2:**
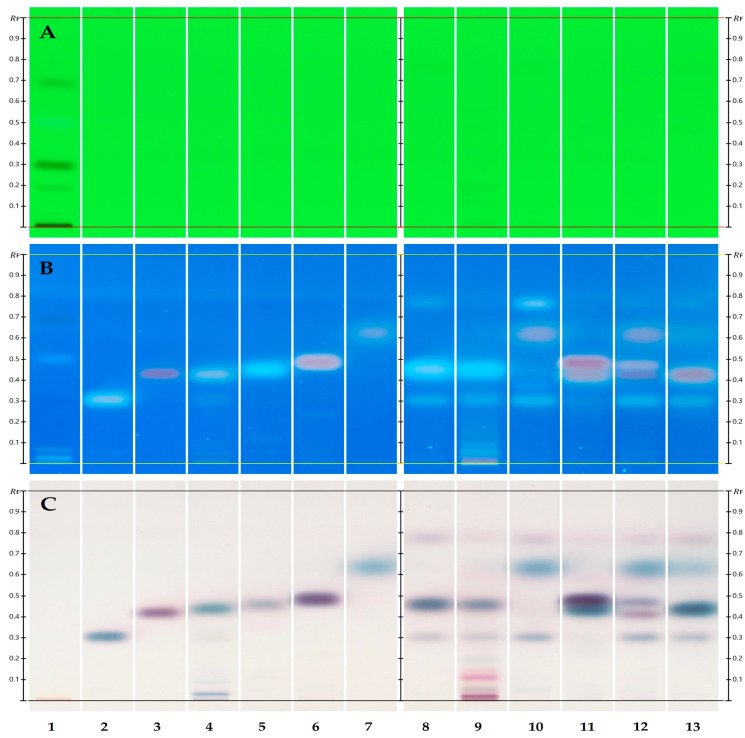
One-dimensional HPTLC separation of TTO components using cyclohexane/ethyl acetate at 8:2 (*v*/*v*). (See Table 1 for track assignment.) Separation of SST before derivatization under shortwave UV light (254 nm) ((**A**) track 1). Reference standards and *Melaleuca* spp. EOs post derivatization under longwave UV (350 nm broadband) (**B**) and white light RT (**C**).

Almost identical results were later obtained with cyclohexane/ethyl acetate at 8:2 (*v*/*v*) as the developing solvent [57] (Figure 2). Cycloxexane, 99+%, CAS # 110-82-7, was obtained from Thermo Fisher Scientific, Ward Hill, MA, USA. Not only is cyclohexane within the same price range as n-hexane, but it is also less hazardous than n-hexane.

**Figure 3:**
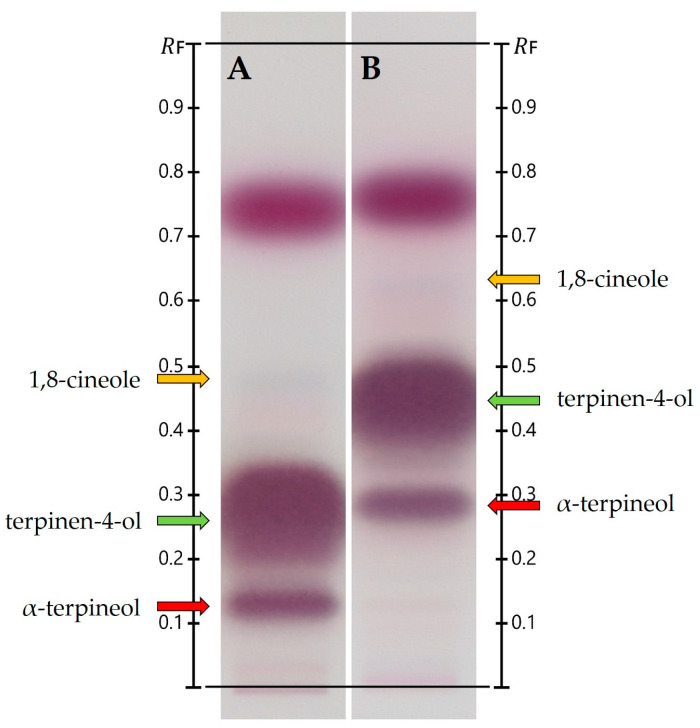
The one-dimensional HPTLC separation of TTO components at higher concentrations (20%) using (**A**) n-hexane/ethyl acetate at 9:1 (*v*/*v*) and (**B**) n-hexane/ethyl acetate at 8:2 (*v*/*v*). Target components are monoterpenes (a non-visible zone with approximate R_F_ between 0.8 and 0.95); sesquiterpenes (purple zone between R_F_ at 0.7 and 0.8); terpinen-4-ol (a wide purple zone marked by a green arrow); and α-terpineol (a narrow purple zone marked by a red arrow). A small percentage of 1,8-cineole can be observed as a narrow blue zone marked by an orange arrow.

The same method was applied at a larger sample concentration (20%) of TTO for the isolation and extraction of target individuals or groups of oil components, for the identification and selection of prospective biologically active compounds (Figure 3).

### 2.2. Two-Dimensional TLC/HPTLC

A 2D approach was attempted to further separate TTO components with similar structures and properties.

#### 2.2.1. Sample and Plate Preparation

Certified tea tree oil was diluted to 20% in methylene chloride. A mobile phase comprising n-hexane/ethyl acetate at an 82:18 (*v*/*v*) ratio was used for both perpendicular separations (Table 2).

#### 2.2.2. Development and Detection

A 20 × 20 cm, 500 μm, glass-backed, silica gel GF UV_254_ Uniplate™ (Miles Scientific, Newark, DE, USA) was activated at 105 °C for 8 min. Ten microliters (10 μL) of 20% TTO was applied at the bottom right of the plate using a CAMAG ATS4 automated sampler. The sample was sprayed as a thin 16 mm long band at 100 nL/s, 20 mm from the bottom and 20 mm from the right edge of the plate.

Manual development was performed in a 20 × 20 cm, single-trough glass chamber (Sigma-Aldrich, St. Louis, MO, USA) containing a 20 × 20 cm #1 chromatography paper (Whatman, Buckinghamshire, UK). Before each development, the chamber was allowed to saturate for 20 min. with the corresponding mobile phase (Table 2) and the plate was quickly placed into the saturated chamber.

Initial development was performed in the traditional position, with a sample application line at the bottom. For the second development (2D), the plate was then turned 90° clockwise and a second development was carried out in this position. Table 1 contains solvent system details. To avoid interference from the remaining mobile phase, the plate was aired out in the fume hood for 7.5 min. after each development.

**Figure 4:**
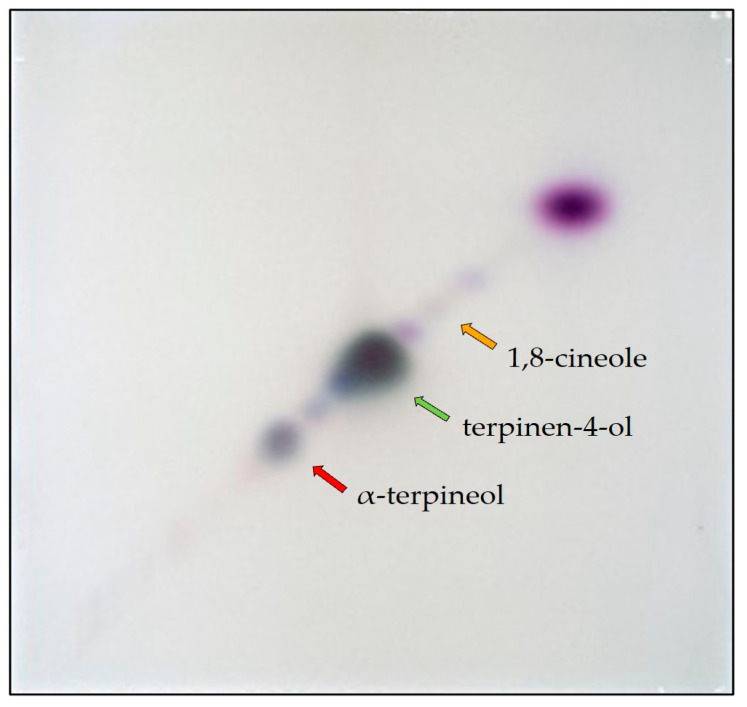
Simple 2D separation of TTO using hexane/ethyl acetate at 82:18 (*v*/*v*) in both directions. α-Terpineol, terpinen-4-ol, and 1,8-cineole are signaled by red, green, and orange arrows, respectively.

Derivatization occurred in a CAMAG derivatizer. A 10 mg/mL vanillin stock was prepared by dissolving 1 g of vanillin in 100 mL 96% ethanol. The derivatizing reagent was then created by adding 80 μL of concentrated sulfuric acid to 4 mL of the ethanolic vanillin solution. The final solution was evenly sprayed onto the plate and colored zones were revealed after heating on a CAMAG Plate Heater III at 105 °C for 1 min. (Figure 4).

A second plate was developed, under the same conditions, but not derivatized. Using the derivatized plate as a template, selected fractions were scraped from the underivatized plate, extracted with n-hexane, and filtered through a 13 mm, 0.2 μm Whatman™ Puradisc 13 (Cytiva, part of Global Life Sciences Solutions USA LLC, Wilmington, DE, USA) using a 3 cc or 5 cc Luer Lok B-D syringe (Becton Dickinson, Franklin Lakes, NJ, USA).

### 2.3. 2D-MGD TLC/HPTLC

A 2D-MGD method was created by adding a multigradient component to the previous 2D method to enhance the separation of α-terpineol from terpinen-4-ol at higher concentrations for isolation and extraction purposes.

#### 2.3.1. Sample and Plate Preparation

A 20% dilution of TTO was prepared in methylene chloride. The mobile phase consisted of n-hexane and ethyl acetate. Four solvent systems of varying polarity were used in sequence to achieve maximum separation (Figure S1, Table 3).

#### 2.3.2. Development and Detection

A 20 × 20 cm, 500 μm, glass-backed, silica gel GF UV_254_ Uniplate^™^ was activated at 100 °C for 10 min, and 8 μL of 20% TTO was applied on the bottom right of the plate using an ATS4 automated sampler. The sample was sprayed as a thin 5 mm long band at 100 nL/s, 20 mm from the bottom and 25 mm from the right edge of the plate. Manual development followed in a 20 × 20 cm, single-trough glass chamber containing a 20 × 20 cm #1 chromatography paper. Before each development, the chamber was saturated with the corresponding mobile phase for 20 min. and the plate was quickly placed into a saturated chamber.

Initial development was performed in the vertical position (0°). The plate was then turned 90° clockwise for the second development and all remaining developments were performed in this perpendicular (90°) position. The plate was aired out in the fume hood for 7.5 min. after each development.

**Figure 5:**
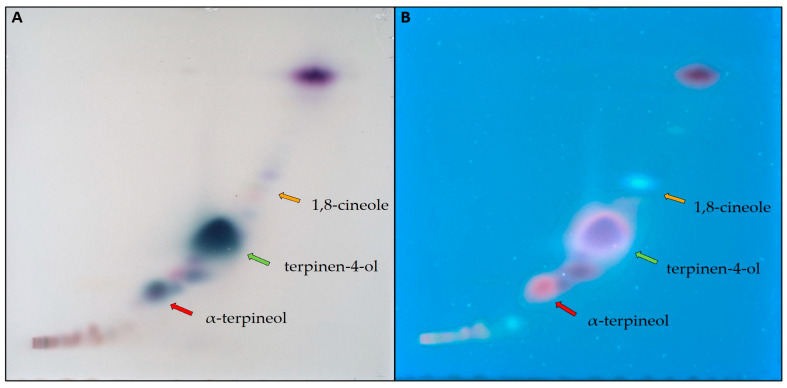
2D-MGD separation of TTO using n-hexane/ethyl acetate multigradient (Table 3) observed under white (**A**) and UV366 (**B**). Color-coded arrows show increased separation of target components.

Derivatization occurred in a CAMAG derivatizer. A reagent was generated by adding 80 μL of concentrated sulfuric acid (H_2_SO_4_) to 4 mL of a 10 mg/mL ethanolic vanillin solution. This reagent was then evenly sprayed onto the plate. Colored zones were revealed after heating the plate at 100 °C for 75 s. (Figure 5).

A second identical plate was developed, but not derivatized. Using the derivatized plate as a template, selected zones were scraped from the underivatized plate and extracted for a further analysis. Extracts were filtered through a 0.2 μm Whatman™ Puradisc 13 connected to a 3 cc or 5 cc Luer Lok syringe.

### 2.4. Gas Chromatography–Mass Spectrometry (GC/MS)

The identification of TTO fractions 1–6 separated by 2D-MGD TLC/HPTLC was performed on a 6890N GC coupled with a 5975B MSD (Agilent Technologies, Inc., Santa Clara, CA, USA). A DB-5 column (30 m × 0.25 mm inner diameter with 0.25 μm film thickness) (Restek Corporation, Bellefonte, PA, USA) was used with a constant helium flow of 1.3 mL/min. The GC oven temperature was kept at 60 °C for 1.3 min and increased to 246 °C at 3 C/min. The temperatures of the ion source and quadrupole were 230 °C and 150 °C, respectively. The mass spectrometry transfer line was 250 °C. Injector and detector temperatures were kept at 220 °C and 230 °C, respectively. MassHunter software, version B.07.06 (Agilent Technologies, Inc., Santa Clara, CA, USA) was used for data acquisition and processing. In total, 1 μL of the diluted sample was injected into the GC-MS on the splitless mode. Mass spectra were recorded at 70 eV, where the mass range was *m*/*z* 35−450 Da with a scan rate of 2.8 scans/s. Relative percentages were directly obtained from peak total ion current (TIC) areas.

#### 2.4.1. Sample Preparation and Fractions

Monoterpene and sesquiterpene hydrocarbon fractions (Fr. 1 and Fr. 2) were obtained from 1D preparatory (prep) TLC separation with a combination of manual and automated 1D TLC. 1,8-Cineole, which comigrated with terpinen-4-ol and monoterpene residuals (Fr. 3), was obtained by the same method.

A Supelco HPTLC plate, silica gel 60G F_254_, and a 20 × 10 cm glass-backed plate (Millipore-Sigma, Burlington, MA, USA) were used as the stationary phase. TTO was diluted to 20% in methylene chloride and the sample was added onto the plate as 40 spots × 2 μL, applied tightly together and forming a line, using a CAMAG ATS4 autosampler equipped with a spot needle. The application line was 8 mm from the bottom edge. A second application immediately followed for a total of 4 μL per spot.

A 1D development was performed manually on a single-trough glass chamber with a 20 × 20 cm Whatman #1 filter paper. The chamber was pre-saturated for 20 min. with 25 mL of n-hexane/ethyl acetate at a ratio of 98:2 (*v*/*v*) as the mobile phase. The solvent front was carried to 85 mm. The developed plate was derivatized in a CAMAG derivatizer with vanillin/H_2_SO_4_ as the derivatizing reagent and heated for 3 min. at 100 °C. A template was created designating TTO fractions according to the colors generated.

A second identical, but underivatized, plate was developed for extraction. Following markings on the previous template, TTO fractions were manually scraped and extracted from the underivatized plate. Fractions were extracted with hexane and filtered using 0.2 μm Whatman Puradisc 13 filters.

Oxygenated monoterpenes (OMs) required further efforts to better separate terpinen-4-ol (Fr. 4) and α-terpineol (Fr. 5), and other oxygenated monoterpenes and sesquiterpenes (Fr. 6). A semi-automated 2D-MGD method (Section 2.3.1) was developed and used for this purpose, and target chemicals were extracted for the GC-MS analysis.

#### 2.4.2. Identification of Components

The identification of TTO fractions 1–6 was conducted by the comparison of their corresponding mass spectra and retention indices (RIs) to those reported in a mass spectral library developed at the USDA-ARS-SHRS laboratory with authentic compounds and with the commercial libraries MassFinder [59], Adams Library [60], Flavours and Fragrances of Natural and Synthetic Compounds 3 (FFNSC-3) [61], and Wiley 12/NIST 2020 [62]. Experimental RIs were calculated using the van Den Dool and Kratz [63] equation to a homologous series of α-alkanes (C9–C21).

### 2.5. Chiral Separation of Terpinen-4-ol and α-Terpineol (GC-FID)

Enantiomeric separation was performed on a 7890B GC equipped with a split/splitless injector (SSL), a flame ionization detector (FID), and OpenLAB software, version 2.3 (Agilent Technologies, Inc., Santa Clara, CA, USA). An Rt-βDEVse chiral capillary column, 30 m × 320 μm × 0.25 mm (Restek, Bellefonte, PA, USA), was installed and helium was supplied at a constant flow of 1.2 mL/min as carrier gas. Using a 10 μL syringe, 1 μL of the sample was introduced into the SSL set at 225 °C and with a split ratio of 10:1. The detector (FID) was maintained at 230 °C.

#### 2.5.1. Sample Preparation

A highly pure terpinen-4-ol extract obtained from 1D prep TLC was diluted at 1:20 in methylene chloride for chiral separation. An α-terpineol sample was obtained by semi-automatic 2D-MGD isolation and used with no further dilution. Identification and purity were confirmed by GC-MSD.

Reference standards of (+)-terpinen-4-ol (CAS# 2438-10-0) and (−)-terpinen-4-ol (CAS# 20126-75-5) were obtained from TCI America, Portland, OR, USA, and Sigma- Aldrich, St. Louis, MO, USA, respectively. (+)-α-Terpineol (CAS# 7785-53-7) and (−)-α-terpineol (CAS# 10482-56-1) were purchased from Sigma-Aldrich, St. Louis, MO, USA. Dilutions of each individual standard, as well as an approximately 1:1 racemic mix of both chemicals, were also prepared at 10 μg/mL in methylene chloride and analyzed under identical conditions to confirm the separation of enantiomers.

#### 2.5.2. Analysis

Two methods were created for the separation of terpinen-4-ol and α-terpineol enantiomers, respectively. All instrument parameters were identical for both, as described at the beginning of this section, apart from the oven temperatures.

**Figure 6:**
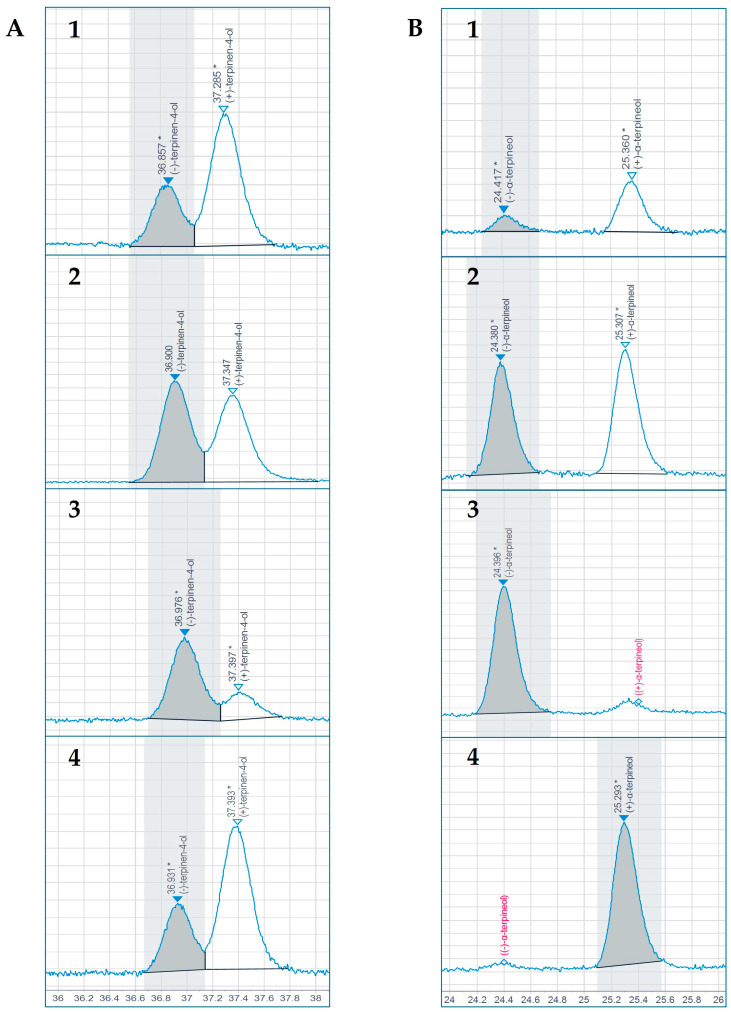
The GC-FID chiral separation of terpinen-4-ol (**A**) and α-terpineol (**B**). In both cases, the enantiomeric distribution is displayed (**1**), followed by a 1:1 reference standard mix (**2**), (−) enantiomer (**3**) and (+) enantiomer (**4**). Asterisk (*) referrs to manual integration.

For terpinen-4-ol chiral separation, the oven was programmed starting at 60 °C and immediately ramping to 95 °C at 5 °C/min. A second ramp to 98 °C at 2 °C/min., followed by a third ramp to 99.7 °C at 0.05 °C/min., separated the enantiomers (Figure 6A). The fourth and final ramp brought the oven to 150 °C at 10 °C/min, holding for 0.47 min. to finalize the analysis.

For α-terpineol, the oven was started at 80 °C with no holding time. A first ramp brought the system to 110 °C at 5 °C/min. This was followed by a second ramp to 112 °C at 2 °C/min. A third ramp to 114 °C at 0.05°C/min. produced the separation of enantiomers (Figure 6B), and a final ramp to 160 °C with a 0.4 min. hold carried the analysis to completion.

## 3. Results

### 3.1. HPTLC Fingerprint/1D TLC

Figure 1 showcases the HPTLC separation of selected *Melaleuca* EOs at a concentration of 1% using n-hexane/ethyl acetate at 8:2 (*v*/*v*) as the mobile phase. Figure 1A highlights the proper separation of the system suitability standard (SST) under UV_254_ (track 1). Three of eight SST zones are visible at R_Fs_~0.18 ± 0.01, 0.27 ± 0.01, and 0.67 ± 0.01, respectively. Figure 1B,C (UV_366_ and RT white light, respectively) display TTO reference standards (Table 1) in ascending migration order followed by samples of certified TTO, aged TTO, and other *Melaleuca* EOs. In Figure 1C, track 8 (certified TTO), terpinen-4-ol, can be observed as the most prominent blue-brown zone at R_F_~0.46, followed by a pink-purple zone containing a mono- and sesquiterpene group at R_F_~0.77, and α-terpineol as a blue zone at R_F_~0.31. An almost imperceptible amount of 1,8-cineole may be seen as a faint blue zone at R_F_~0.64.

Aged TTO (Figure 1C, track 9), as well as the presence of other *Melaleuca* EOs (Figure 1C, tracks 10 (Cajeput), 11 (Nerolina), 12 (Niaouli), and 13 (Rosalina)), can be identified by a lower amount or absence of terpinen-4-ol, an increased amount of 1,8-cineole, or the presence of different prominent components.

Results obtained in a parallel analysis using cyclohexane/ethyl acetate at 8:2 (*v*/*v*) as the mobile phase (Figure 2) were almost identical to those with n-hexane/ethyl acetate at 8:2 (*v*/*v*) (Figure 1). No significant variation in R_F_ values was observed on the SST, reference standards, or EO samples.

At higher TTO concentration (20%), using a 9:1 ratio of n-hexane and ethyl acetate (Figure 3A) revealed that a good separation of 1,8-cineole (marked by orange arrows) may interfere with the separation of terpinen-4-ol (green arrows) and α-terpineol (red arrows). With n-hexane/ethyl acetate at 8:2 (*v*/*v*) as the developing solvent (Figure 3B), the case seemed to reverse. Terpinen-4-ol and α-terpineol achieved better separation; yet, 1,8-cineole merged with neighboring compounds.

### 3.2. Two-Dimensional TLC/HPTLC

On a semi-automated 2D-TLC/HPTLC system using the same mobile phase (n-hexane/ethyl acetate at 82:18 (*v*/*v*)) for both perpendicular separations (Table 2), a symmetrical diagonal pattern was obtained (Figure 4). Higher resolution is evident between the three components of interest, 1,8-cineole, terpinen-4-ol, and α-terpineol, as marked by corresponding color-coded arrows. However, new compounds can also be observed in the vicinity of our target compounds that were not seen with 1D TLC/HPTLC due to converging R_F_s.

### 3.3. 2D-MGD TLC/HPTLC and Characterization of Fractions

The successful separation of TTO using 2D TLC and 2D-MGD, in combination with HPTLC, was confirmed via GC-MS (Table 4, Figure S2). Results show that a semi-automated multigradient development resulted in some evaporation of mono- (Fr. 1) and sesquiterpene hydrocarbons (Fr. 2); yet, it yielded 1.87% 1,8-cineole among some terpinen-4-ol and monoterpene residuals (Fr. 3), 100% terpinen-4-ol (Fr. 4), and 94.06% α-terpineol (Fr. 5). Fraction 6 was rich in oxygenated monoterpenoids (50.41%) and oxygenated sesquiterpenoids (45.48%).

### 3.4. Enantiomeric Distribution of Terpinen-4-ol and α-Terpineol

Fractions obtained from 2D-MGD TLC/HPTLC separations had proven to be highly pure after the GC-MS analysis. Positive and negative enantiomers of each chemical were confirmed by individual enantiomer reference standards, and by a racemic mix of each, analyzed under identical conditions (Figure 6).

Our terpinen-4-ol samples, obtained by semi-automated 2D-MGD extraction, yielded an average of 69.44% positive (+) enantiomers (by area) with an absolute (+)/(−) ratio of 2.27, whereas α-terpineol yielded 77.36% (+) enantiomers and an absolute (+)/(−) ratio of 3.42 (Table 5).

## 4. Discussion

### 4.1. HPTLC Fingerprint/1D TLC

It has been established [19] that a large amount (35–48%) of terpinen-4-ol, a noticeable amount (2–5%) of α-terpineol, and a low amount (trace—10%) of 1,8-cineole characterize certified TTO. The chemical composition of TTO was also reported in a recent HPTLC application note [57]. In this application note, a fully automated HPTLC fingerprint of TTO at lower concentration (1%) was created that follows the pharmacopeia protocol and confirms amounts of key ingredients. Observed retention factors and amounts are comparable with those in the literature.

Decay and adulteration are major factors to consider for quality control of EOs since they alter the chemical composition of the oil [18,64]. The accurate separation and quantification of key ingredients are imperative for the quality assurance of the commercial product, not only for TTO but also for other EOs as well. An accurate example is recent HPTLC work by HPTLC Association that defines the chemical composition of Niaouli (*M. quinquenervia* (Cav.), S.T. Blake) EO [65]. In this work, Niaouli EO was separated using toluene/ethyl acetate at 95:5 (*v*/*v*) and also compared with corresponding reference standards and other *Melaleuca* EOs to confirm authenticity. The current TTO fingerprint was created using the same SOP with the exception of the mobile phase. In this case, toluene was substituted with n-hexane and later cyclohexane in the solvent system as more ecologically friendly solvents.

For some experiments, higher amounts of isolated oil components may be required to be utilized in tandem studies or collaborative research. This poses an added challenge in achieving the necessary separation for the extraction of individual TLC zones. More recent HPTLC work [66] showcased a 1D HPTLC separation of TTO at a higher concentration (20%) with two different solvent systems. A mobile phase comprising n-hexane/ethyl acetate at 9:1 (*v*/*v*) offered a better separation of less polar, more volatile mono- and sesquiterpene hydrocarbons in the upper R_F_ region; yet, more polar oxygenated monoterpenes at lower R_F_ did not separate to a full extent (Figure 3A). Changing the solvent ratio to n-hexane/ethyl acetate at 8:2 (*v*/*v*) provided a sharper separation over a wider R_F_ range, improving the separation of terpinen-4-ol and α-terpineol (Figure 3B). Green and red arrows mark the change in R_F_ for terpinen-4-ol and α-terpineol, respectively.

In the case of 1,8-cineole, TLC separation and quantification proved to be difficult due to its minor presence in the oil and its tendency to diffuse. Orange arrows revealed that a good separation of 1,8-cineole with hexane/ethyl acetate at 9:1 (*v*/*v*) may interfere with the separation of terpinen-4-ol and α-terpineol in the oil (Figure 3A). With hexane/ethyl acetate at 8:2 (*v*/*v*) as the developing solvent (Figure 3B), the case reversed. Terpinen-4-ol and α-terpineol achieved better separation, while the GC-MS analysis showed that 1,8-cineole merges with neighboring compounds. A semi-automated two-dimensional approach was then investigated to further improve the separation of terpinen-4-ol, α-terpineol, and 1,8-cineole in a single sample.

### 4.2. Two-Dimensional TLC/HPTLC

Due to limitations in the automatic developing chamber’s size and related processing capabilities, a fully automated method could not be used for this portion of the study. The module is capable of holding plates up to 20 × 10 cm and the experiment required a larger plate size (20 × 20 cm) for larger extraction purposes. Therefore, a hybrid manual/automated protocol was developed to perform 2D-TLC separations. Plate development was conducted manually, while all other steps were automated to reduce human error and increase reproducibility.

Using the same mobile phase for both perpendicular separations (hexane/ethyl acetate at 82:18 (*v*/*v*)) (Table 2), a symmetrical diagonal pattern was obtained (Figure 4). A higher resolution is evident between the three components of interest, α-terpineol, terpinen-4-ol, and 1,8-cineole, when developed using 2D-TLC, as marked by the corresponding color-coded arrows. However, new surrounding compounds were also revealed that were not seen with traditional 1D TLC/HPTLC due to converging RFs. Proper separation seemed to require a wider polarity gradient than that of a simple 2D development.

### 4.3. 2D-MGD TLC/HPTLC and Characterization of Fractions

Following the semi-automatic protocol used for simple 2D TLC, a 2D-MGD method was developed using a series of n-hexane/ethyl acetate ratios in sequence as described in Table 3. An initial general separation was achieved with an 85:15 (*v*/*v*) ratio. The plate was then turned 90 degrees clockwise and re-developed three more times starting with a 99:1 (*v*/*v*) ratio, followed by 97:3 and ending with 95:5. This method increased the separation of our components of interest from other surrounding components. Figure 5 shows an enhanced separation of three target chemicals marked by arrows.

When carrying out multigradient separations, time is of the essence to avoid the evaporation of more volatile compounds. This was another reason to opt for manual development. Since software control of the automatic chamber calls for the plate to be placed inside the chamber during the saturation and activation process, it results in the evaporation of our more volatile components of interest. Using multiple manual glass chambers, which did not require the plate to be inside for saturation, that step could be overlapped to shorten the process and reduce evaporation.

### 4.4. Gas Chromatography–Mass Spectrometry

The GC-MS analysis confirmed the successful separation of TTO into six fractions using polarity-based solvent ratios of n-hexane/ethyl acetate by incorporating 1D- and 2D-TLC/HPTLC and 2D-MGD TLC/HPTLC methods. The polarity-based fractionation of TTO provided a clear separation of hydrocarbons from oxygen-containing terpenoids. The representative chromatograms are given in Figure S2. The first and second fractions comprised monoterpene and sesquiterpene hydrocarbons. The subsequent three fractions (fractions 3 to 5) contained oxygenated monoterpenoids. The polar compounds present in the TTO remained in the baseline area on the TLC plate. Fraction 6 was dominated by a series of oxygenated sesquiterpenoids. Succinctly, combined TLC/HPTLC protocols proved to be quite useful in providing the rapid separation of TTO into its major chemical classes.

### 4.5. Enantiomeric Distribution of Terpinen-4-ol and α-Terpineol

The enantiomeric ratio of each, terpinen-4-ol and α-terpineol, in TTO has been previously established and has been incorporated into the ISO criteria for its authentication. It has been reported [55,56] that the positive (+) enantiomer exists in a significantly higher amount than the (−) enantiomer for both chemicals, with an absolute enantiomeric range between 67.4 and 69.6% for terpinen-4-ol, and 71.0 and 78.0% for α-terpineol, respectively. Authors also reported absolute enantiomeric (+)/(−) ratios ranging between 2.07 and 2.23 for terpinen-4-ol, and between 2.45 and 3.54 for α-terpineol [55,56]. Absolute values and ratios obtained in this study by chiral GC-FID (Table 5) fell slightly above but are still consistent with those reported in the literature.

### 4.6. Discussion Summary

In summary, the 2D-MGD TLC/HPTLC method proved to be more efficient than other single techniques in this study for the separation of TTO components in relatively large amounts to be used for further research. The most notable advantage is the total isolation of our target OMs, terpinen-4-ol and α-terpineol. Another benefit is an improved separation and decreased evaporation of monoterpene and sesquiterpene hydrocarbons.

Nonetheless, there are some limitations to the procedure. The first limitation is the human error introduced by performing manual development. Another limitation arises from the high volatility of mono- and sesquiterpenes, which prompts us to use multiple manual chambers to carry out multigradient developments. To avoid total loss of volatile components, a chamber for the following step must be saturated while the previous development is still in progress. In addition, monoterpenes provide the challenge of not producing a color change when derivatized. Hence, there is the need for a GC-MS analysis for confirmation. However, once confirmed once, GC-MS validation is no longer necessary unless the 2D-MGD TLC/HPTLC protocol is altered.

## 5. Conclusions

The results of this study revealed that HPTLC is applicable for the screening of TTO for authenticity and stability. Full automation minimizes human error, and it is also useful for larger amounts or higher concentrations of samples, with minor modifications. HPTLC has several advantages over TLC: (i) enhanced reproducibility, (ii) improved separation of monoterpene and sesquiterpene hydrocarbons, (iii) shorter separation time, (iv) lower solvent volume, and (v) 2D-MGD TLC/HPTLC allowed a higher purification of coeluting compounds such as terpinen-4-ol and α-terpineol. To achieve finer separation, more complex methodology is required. Further research is needed to achieve the isolation of individual lipophilic components, difficult to separate by 1D and 2D TLC.

Semi-automated, single-gradient 2D-TLC improved the separation of monoterpenes and sesquiterpenes on the upper R_F_ region. Manual development allowed for the use of larger, thicker plates and reduced the evaporation of the more volatile chemicals. The separation of terpinen-4-ol and α-terpineol, as well as 1,8-cineole, required a stronger solvent gradient. The 2D-MGD separation of TTO resulted in a highly pure extraction of these two similar oxygenated monoterpenes.

The accurate identification and quantification of TLC- and HPTLC-isolated chemicals, or groups of components, was confirmed by GC-MS. The enantiomeric distributions of terpinen-4-ol and α-terpineol, obtained from TLC/HPTLC separation and quantified by GC-FID, were within the range belonging to pharmacopeia and ISO requirements. Fully and semi-automated 2D-MGD allows for simultaneous analyses of up to 15 samples under identical conditions, which makes it an excellent technique for high-throughput analyses. More so, due to the system’s reproducibility, once confirmed by GC-FID and GC-MS, the TLC/HPTLC method may be deemed reliable as a standalone technique, if method parameters are not altered.

Further research is required to attempt to overcome the limitations of the procedure and to achieve an enhanced separation of other TTO components of interest.

## Figures and Tables

**Table 1:** HPTLC track descriptions (Figure 1 and Figure 2).

Track	Name	CAS #	Source
1	UHM	---	Sigma- Aldrich, St. Louis, MO, USA
2	(−)-α-terpineol	10482-56-1	Sigma- Aldrich, St. Louis, MO, USA
3	viridiflorol	0552-02-03	Sigma- Aldrich, St. Louis, MO, USA
4	(−)-linalool	1126-91-0	Sigma- Aldrich, St. Louis, MO, USA
5	(−)-terpinen-4-ol	20126-76-5	Sigma- Aldrich, St. Louis, MO, USA
6	nerolidol	7212-44-4	Sigma- Aldrich, St. Louis, MO, USA
7	1,8-cineole	470-82-6	Sigma- Aldrich, St. Louis, MO, USA
8	*M. alternifolia* (Maiden & Betche) Cheel (tea tree oil)	---	Apothecary Shoppe, Portland, OR, USA
9	*M. alternifolia* (Maiden & Betche) Cheel (tea tree oil)	---	SAT Group, Kannauj, India
10	*M. cajuputi* Powell (Cajeput oil)	---	Nature’s Gift, Madison, TN, USA
11	*M. quinquenervia* (Cav.) S.T.Blake linalool CT (Nerolina oil)	---	Nature’s Gift, Madison, TN, USA
12	*M. quinquenervia* (Cav.) S.T.Blake cineole CT (Niaouli oil)	---	Nature’s Gift, Madison, TN, USA
13	*M. ericifolia* Sm. (Rosalina oil)	---	Nature’s Gift, Madison, TN, USA

**Table 2:** Development of solvent systems for 2D separation of TTO.

Development Number	Solvent System	Vol, mL	Ratio	Solvent Front, cm	Direction
1	n-hexane/ethyl acetate	50	82:18	18	0° (traditional position)
2	n-hexane/ethyl acetate	50	82:18	18	90° clockwise

**Table 3:** The sequence of mobile phases used for 2D-MGD TLC of TTO.

Development Number	Solvent System	Vol, mL	Ratio	Solvent Front, cm	Direction
1	n-hexane/ethyl acetate	50	85:15	18	0° (traditional position)
2	n-hexane/ethyl acetate	50	99:1	18	90° clockwise
3	n-hexane/ethyl acetate	50	97:3	17	Remain at 90°
4	n-hexane/ethyl acetate	50	95:5	15	Remain at 90°

**Table 4:** 2D-TLC and 2D-MGD separation of TTO fractions 1–6.

* RI Exp	** RI Lit	Compounds	Total TTO	Fr-1	Fr-2	Fr-3	Fr-4	Fr-5	Fr-6
938	930	α-Thujene ^RI, MS^	0.09	-	-	-	-	-	-
946	939	α-Pinene ^RI, MS, Std^	3.37	0.11	-	-	-	-	-
970	975	Sabinene	0.05	-	-	-	-	-	-
989	979	β-Pinene ^RI, MS, Std^	0.17	0.44	0.26	-	-	-	-
998	990	Myrcene ^RI, MS, Std^	0.64	0.24	0.13	-	-	-	-
1010	1002	α-Phellandrene ^RI, MS, Std^	0.58	0.54	0.21	0.03	-	-	-
1022	1017	α-Terpinene ^RI, MS, Std^	10.7	27.39	6.57	1.39	-	-	-
1030	1024	*p*-Cymene ^RI, MS, Std^	1.0	3.24	1.19	0.12	-	-	-
1039	1029	Limonene ^RI, MS, Std^	0.5	0.71	0.06	-	-	-	-
1039	1029	β-Phellandrene ^RI, MS, Std^	0.38	-	0.04	-	-	-	-
1040	1031	1,8-Cineole ^RI, MS, Std^	2.29	-	-	1.87	-	-	-
1071	1059	γ-Terpinene ^RI, MS, Std^	19.79	57.94	17.75	3.20	-	-	-
1081	1070	*cis*-Sabinene hydrate ^RI, MS^	0.01	-	-	-	-	3.09	-
1084	1072	*cis*-Linalool oxide ^RI, MS, Std^	-	-	-	-	-	-	-
1098	1088	Terpinolene ^RI, MS, Std^	3.63	9.39	5.12	0.76	-	-	-
1107	1096	Linalool ^RI, MS, Std^	0.12	-	-	0	-	-	0.57
1123	1121	*cis*-*p*-Menth-2-en-1-ol	0.28	-	-	0	-	-	8.15
1125	1122	*trans*-*p*-Menth-2-en-1-ol	0.19	-	-	0	-	-	4.93
1183	1177	Terpinen-4-ol ^RI, MS, Std^	37.95	-	-	91.53	100	-	-
1188	1182	*p*-Cymen-8-ol ^RI, MS^	-	-	-	-	-	-	5.65
1198	1188	α-Terpineol ^RI, MS, Std^	3.12	-	-	0.98	-	94.06	1.38
1206	1196	*cis*-Piperitol ^RI, MS, Std^	0.07	-	-	-	-	-	0.68
1218	1208	*trans*-Piperitol ^RI, MS^	0.1	-	-	-	-	-	4.94
1273	1269	*trans*-Ascaridol glycol ^RI, MS^	0.02	-	-	-	-	-	24.11
1291	1288	*cis*-Ascaridol glycol ^RI, MS^	0.01	-	-	-	-	-	-
1356	1348	α-Cubebene ^RI, MS^	0.05	-	0.50	-	-	-	-
1378	1376	Isoledene ^RI, MS^	0.09	-	0.48	-	-	-	-
1380	1376	α-Copaene ^RI, MS, Std^	0.16	-	1.00	-	-	-	-
1395	1390	β-Elemene ^RI, MS, Std^	0.02	-	0.23	-	-	-	-
1410	1409	α-Gurjunene ^RI, MS^	0.61	-	2.87	-	-	-	-
1417	1416	β-Maaliene ^RI, MS^	0.03	-	0.25	-	-	-	-
1421	1419	β-Caryophyllene ^RI, MS, Std^	0.77	-	4.33	-	-	-	-
1429	1425	γ-Maaliene ^RI, MS^	0.09	-	0.68	-	-	-	-
1435	1433	α-Maaliene ^RI, MS, Std^	0.05	-	0.71	-	-	-	-
1439	1441	Aromadendrene ^RI, MS, Std^	1.90	-	10.24	-	-	-	-
1443	1443	Selina-5,11-diene ^RI, MS^	0.23	-	1.42	-	-	-	-
1450	1453	*trans*-Muurola-3.5-diene ^RI, MS^	0.21	-	1.30	-	-	-	-
1453	1454	α-Humulene ^RI, MS, Std^	0.13	-	0.85	-	-	-	-
1459	1460	Alloaromadendrene ^RI, MS, Std^	0.99	-	5.73	-	-	-	-
1473	1476	*trans*-Cadina-1(6),4-diene ^RI, MS^	0.58	-	3.55	-	-	-	-
1476	1479	γ-Muurolene ^RI, MS^	0.05	-	0.34	-	-	-	-
1486	1490	β-Selinene ^RI, MS^	0.12	-	0.90	-	-	-	-
1488	1490	Alloaromadendr-9-ene ^RI, MS^	0.30	-	0.90	-	-	-	-
1492	1493	*cis*-β-Guaiene ^RI, MS^	0.12	-	1.76	-	-	-	-
1494	1496	Ledene ^RI, MS^	2.02	-	11.69	-	-	-	-
1497	1500	Bicyclogermacrene ^RI, MS^	0.43	-	1.98	-	-	-	-
1498	1500	α-Muurolene ^RI, MS^	0.05	-	0.65	-	-	-	-
1501	1501	Epizonarene ^RI, MS^	0.04	-	0.20	-	-	-	-
1512	1513	γ-Cadinene ^RI, MS^	0.02	-	0.37	-	-	-	-
1518	1522	*trans*-Calamene ^RI, MS^	0.01	-	0.25	-	-	-	-
1521	1523	δ-Cadinene ^RI, MS^	2.27	-	11.74	-	-	-	-
1527	1529	Zonarene ^RI, MS^	0.06	-	1.02	-	-	-	-
1531	1534	*trans*-Cadina-1,4-diene	0.29	-	1.85	-	-	-	-
1535	1538	α-Cadinene ^RI, MS^	0.01	-	0.23	-	-	-	-
1560	1563	(*E*)-Nerolidol ^RI, MS, Std^	0.07	-	-	-	-	-	1.31
1562	1567	Maaliol ^RI, MS^	0.02	-	-	-	-	-	0.96
1563	1568	Palustrol ^RI, MS^	0.09	-	-	-	-	-	0.92
1572	1578	Spathulenol ^RI, MS^	0.09	-	-	-	-	-	18.85
1585	1590	Globulol ^RI, MS, Std^	0.47	-	-	-	-	-	6.90
1586	1592	Viridiflorol ^RI, MS, Std^	0.18	-	-	-	-	-	1.81
1588	1595	Cubeban-11-ol ^RI, MS^	0.18	-	-	-	-	-	1.63
1594	1600	Rosiflorol ^RI, MS^	0.14	-	-	-	-	-	5.15
1597	1600	Guaiol ^RI, MS^	0.15	-	-	-	-	-	1.58
1599	1602	Ledol ^RI, MS^	0.24	-	-	-	-	-	1.58
1609	1607	5-e*pi*-7-*epi*-α-Eudesmol ^RI, MS^	0.12	-	-	-	-	-	4.79
		Total	98.51	100	99.35	99.88	100	97.15	95.89

* RI Lit: RI from the literature [MassFinder, Adams, FFNSC3, Wiley, van Den Dool] [59,60,61,62,63]; ** RI Exp: Retention indices (RIs) calculated from the current study [63]. Identification method—^RI^: retention index; ^MS^: computer matching of the mass spectra libraries and comparison with literature data; ^Std^: compounds were purchased.

**Table 5:** Experimental enantiomeric distribution of terpinen-4-ol and α-terpineol by GC-FID.

Chemical	(+) Enantiomer (n = 3)	(−) Enantiomer (n = 3)	(+)/(−) Ratio (n = 3)
terpinen-4-ol	69.4 ± 0.6	30.6 ± 0.6	2.27 ± 0.06
α-terpineol	77.4 ± 0.2	22.6 ± 0.2	3.42 ± 0.04

## Data Availability

Data are contained within the article.

## Supplementary Materials

The following supporting information can be downloaded at: https://www.mdpi.com/article/10.3390/biom15010147/s1, Figure S1. The 2D-MGD method diagram describes the plate after sample application (A), first (1D) development (B), 90° plate rotation (C), and sequential multigradient developments in the second dimension (2D) (D–F). Figure S2. Chromatograms of TTO fractions 1–6 identified by GC-MS.
