# Supplementary material for: Development of a Combined 2D-MGD TLC/HPTLC Method for the Separation of Terpinen-4-ol and α-Terpineol from Tea Tree, Melaleuca alternifolia, Essential Oil
Source: Biomolecules. 2025 Jan 18;15(1):147. doi: 10.3390/biom15010147 (PMC11764153; doi:10.3390/biom15010147)
Supplement: Supplementary file 1 [file biomolecules-15-00147-s001.zip › biomolecules-3381938-supplementary.pdf]

Supplementary Materials:

Figure S1. 2D-MGD method diagram describes the plate after sample application (A), first (1D) development (B), 90° rotation, and sequential multi-gradient developments in the second dimension (2D) (D-F).

Figure S2. Chromatograms of TTO fractions 1-6 identified by GC-MS.

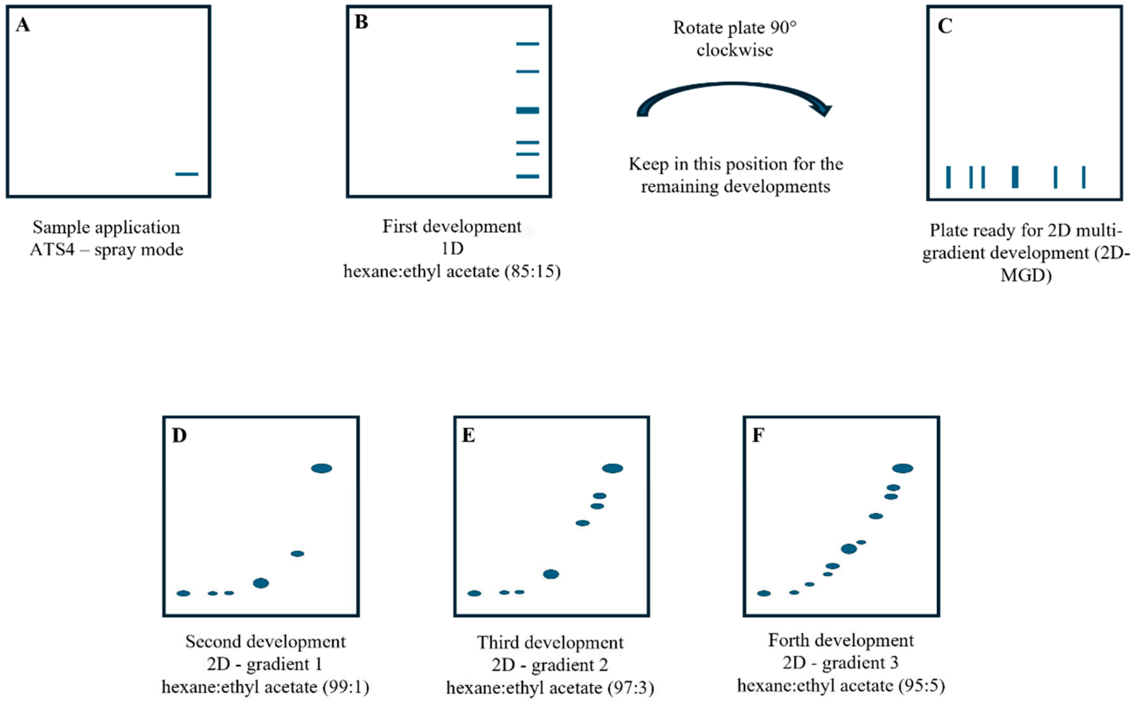

Figure S1. 2D-MGD method diagram describes the plate after sample application (A), first (1D) development (B), 90° plate rotation (C), and sequential multi-gradient developments in the second dimension (2D) (D – F).

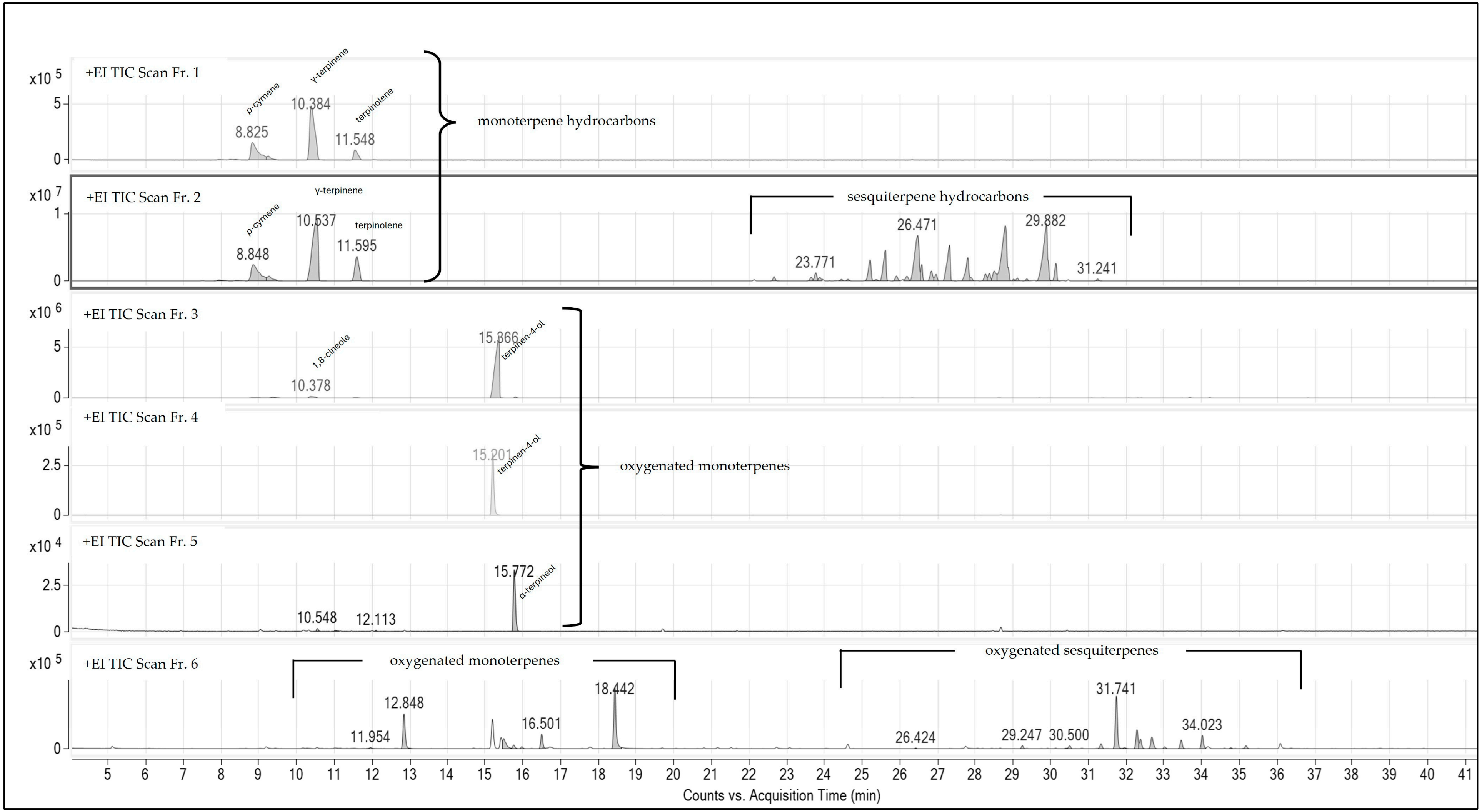

Figure S2: Chromatograms of TTO fractions 1-6 identified by GC-MS.
